# Local electricity pricing across Sweden: data and method

**DOI:** 10.1016/j.dib.2026.113191

**Published:** 2026-08-28

**Authors:** Gabriella Grenander, David Daniels

**Affiliations:** The Swedish National Road and Transport Research Institute, Regnbågsgatan 1, 417 55 Gothenburg, Sweden

**Keywords:** DSO, Power grid tariffs, Network fee structures, Transmission and distribution costs

## Abstract

This dataset provides hourly commercial electricity prices for all locations in Sweden for 2019–2024; all prices are nominal. It combines wholesale market prices with local distribution network power tariffs in a reproducible processing pipeline. Wholesale electricity prices originate from the Nord Pool day‑ahead market and vary hourly in each of Sweden’s four bidding areas (SE1–SE4). Grid tariffs, by contrast, are determined by the local DSOs responsible for operating Sweden’s electricity distribution grids. As of 2025, 145 DSOs operate within 310 non‑overlapping network concession areas in Sweden, and each may determine distinct tariff structures. Grid tariff data were imported from the Swedish Energy Markets Inspectorate’s (EI’s) reporting entity (RE/REL) tables, covering fixed fees, energy-based charges, subscribed-capacity components, and high-load definitions for capacity-based commercial customers. Additional tariff attributes not explicitly encoded in EI’s workbook—such as high-load months and hours or capacity definitions—were collected from DSO websites and compiled in a structured JSON file. All data were cleaned, unit-normalized, time-aligned and organized using Python.

The resulting dataset includes hourly wholesale prices, harmonized tariff components, machine-readable tariff rules. The processing framework accepts either 24-hour template load profiles (expanded automatically to annual resolution) or full 8760-hour load profiles, enabling analysis that preserves seasonal and temporal variation.

The resource is designed to support reproducible electricity cost modeling, tariff structure evaluation, and scenario-based analyses of temporal and spatial variation in end user costs. All inputs, scripts, and outputs are openly provided to enable flexible reuse for different load profiles, study areas, and future data extensions.

Specifications Table

**Table:** 

Subject	Earth & Environmental Sciences
Specific subject area	Wholesale and commercial retail electricity price data within Sweden for the years 2019–2024, suitable for inclusion in economic models of the Swedish electric power network or energy system
Type of data	Type of data: Tabular data, processed data, raw data, scripts and code Formats: CSV (processed outputs), Excel (raw input files), PNG/JPEG (visual aids), Python (.py scripts)
Data collection	Hourly wholesale prices (2019–2024) were downloaded from Vattenfall for bidding areas SE1–SE4. Grid tariff data were imported from EI’s RE/REL-indexed Excel files. Additional grid tariff data (subscribed capacity definitions, high load hours etc.) was collected from DSO websites. All inputs were cleaned, unit-normalized, and structured using Python, including timestamp parsing and construction of annual hourly series for cost modeling.
Data source location	Country: Sweden
Data accessibility	Repository name: Electricity_Cost_Calculator-Sweden Data identification number: 10.5281/zenodo.18,924,687 Direct URL to data: https://github.com/GabriellaGrenanderVTI/Electricity_Cost_Calculator-Sweden
Related research article	G. Grenander, D. Daniels, Price Signals and Load Flexibility in Public Transport: A Cost Analysis of Electric Bus Charging, Findings (2026) [1]

## 1 Value of the Data

• **Provides consolidated historical retail electricity pricing data for Sweden:** This dataset compiles hourly wholesale electricity prices for Sweden’s four bidding areas (SE1–SE4) and combines them with detailed grid tariff structures across all Swedish distribution system operators (DSOs) for 2019–2024. Wholesale prices originate from Nord Pool and are distributed across multiple platforms and formats (e.g., ENTSO-E or, historically, Vattenfall). Grid tariffs, published by the Swedish Energy Markets Inspectorate (EI), are available at the reporting‑entity level but require significant processing to become useful for further analysis. By consolidating these sources into a coherent structure with standardized units and timestamps, and by incorporating DSO-specific tariff rules that are not available in machine-readable form, the dataset eliminates manual pre‑processing work for researchers.
  Many studies rely on wholesale electricity prices alone or approximate retail prices using simplified markups. However, end-user electricity costs depend on both wholesale prices and local network tariffs, which can vary considerably between DSOs. The dataset therefore provides a more realistic basis for studying commercial end‑user electricity costs, demand‑side flexibility, transport electrification, or spatiotemporal variation in electricity price signals across Sweden.
• **Captures the complexity of Sweden’s electricity grid tariff system:** Many electricity market and energy system studies focus primarily on wholesale electricity prices and approximate retail prices using simple markups or average network charges. While such simplifications are often necessary at large geographic scales, they do not capture the variation introduced by local distribution network tariffs. In Sweden, network costs can be of similar magnitude to wholesale electricity costs and may vary considerably between DSOs, even within the same bidding or municipal area [1].
  The data reflect the three‑level structure of Swedish network costs—national transmission, regional distribution, and local distribution—while providing tariff information across all Swedish network concession areas and local DSOs. DSOs design their own tariff structures, which should reflect local network conditions and maintenance costs. The diversity of tariff formats, subscribed capacity definitions and high-load time definitions across DSOs are preserved, offering a transparent foundation for studying spatial variability, tariff design, and regulatory environments without needing to reconstruct each DSO’s pricing scheme manually.
  Detailed retail electricity price datasets are rare because local tariff structures vary substantially between DSOs. By preserving this diversity rather than replacing it with simplified average tariffs, the dataset enables direct comparison of electricity costs and price signals across Swedish regions under identical assumptions.
• **Supports custom scenario analysis based on user-defined load profiles:** The dataset includes tools that allow users to upload either a 24-hour representative profile, which the system expands to 8760 hours with weekday/weekend and seasonal logic, or a full 8760-hour load profile. The latter allows direct analysis of seasonal variations. Costs are then calculated by applying both wholesale prices and all tariff components, including those requiring load information to compute, such as subscribed power or high‑load charges. This enables analysis ranging from small time‑of‑day load adjustments to full-year operational scenarios for applications such as electric vehicle fleets, building energy systems, and industrial demand response studies. Example load profiles and workflows are included to facilitate ease of use and reproducibility.
• **Enables policy, tariff design, and system-level research:** The dataset is structured to support evaluations of how tariff structures affect consumer costs and the impacts of demand flexibility. It supports simulations of tariff reforms, such as capacity-based charges or dynamic pricing, and provides evidence for evaluating equity and efficiency in electricity pricing across regions.
• **Structured for modeling and integration into analytical workflows:** All data are provided in standardized formats (CSV, Excel, JSON), and the accompanying Python modules for preprocessing, load profile expansion, and cost calculation are included, making the dataset suitable for integration into energy system models and optimization frameworks. The workflow structure enables extension to future years and inclusion of new data sources, supporting long‑term use.

## 2 Background

This dataset was developed to support analyses that require linking hourly electricity consumption to both wholesale energy prices and local grid tariff structures in Sweden. Wholesale electricity prices vary hourly across the four Nord Pool bidding areas (SE1–SE4), while local grid tariffs may differ across the 145 distribution system operators (DSOs) responsible for Sweden’s 310 network concession areas (as of 2025). Because end-user electricity costs depend on both the temporal load profile and the specific tariff structure of the local DSO, integrated and time-resolved price data are necessary for electricity cost modeling. With Sweden requiring its DSOs to introduce time-differentiated power tariffs beginning in 2027 [2], this dataset makes a timely contribution that facilitates independent tariff design, impact studies, and other types of fact-based decision support and evaluation.

The dataset combines hourly wholesale prices for 2019–2024 with tariff components reported at the reporting-entity level (RE/REL) by the Swedish Energy Markets Inspectorate. Although the underlying data originate from public sources, substantial processing was required before they could be used for electricity cost analysis. In particular, information describing how DSOs define subscribed capacity charges, peak determination, high-load definitions, and time-restricted pricing windows was not available in a standardized machine-readable format and was therefore collected manually from DSO documentation and websites. The tariff rules were subsequently harmonized and encoded into a common JSON schema, allowing diverse tariff structures to be applied to hourly load profiles with consistency. The resulting dataset integrates fixed, energy-based, and power-based tariff components with the supplementary tariff logic to calculate end-user electricity costs.

This data article also supports the related research article *“Price Signals and Load Flexibility in Public Transport: A Cost Analysis of Electric Bus Charging”* [1], in which annual electricity costs were computed for charging scenarios of an electric-bus fleet in Skövde. By publishing the consolidated price data, tariff structures, and Python-based workflow used in that study, this article enables others to reproduce the underlying methodology and apply it to different load profiles, study areas, and years.

## 3 Data Description

### 3.1 Repository structure

**Fig. 1:**
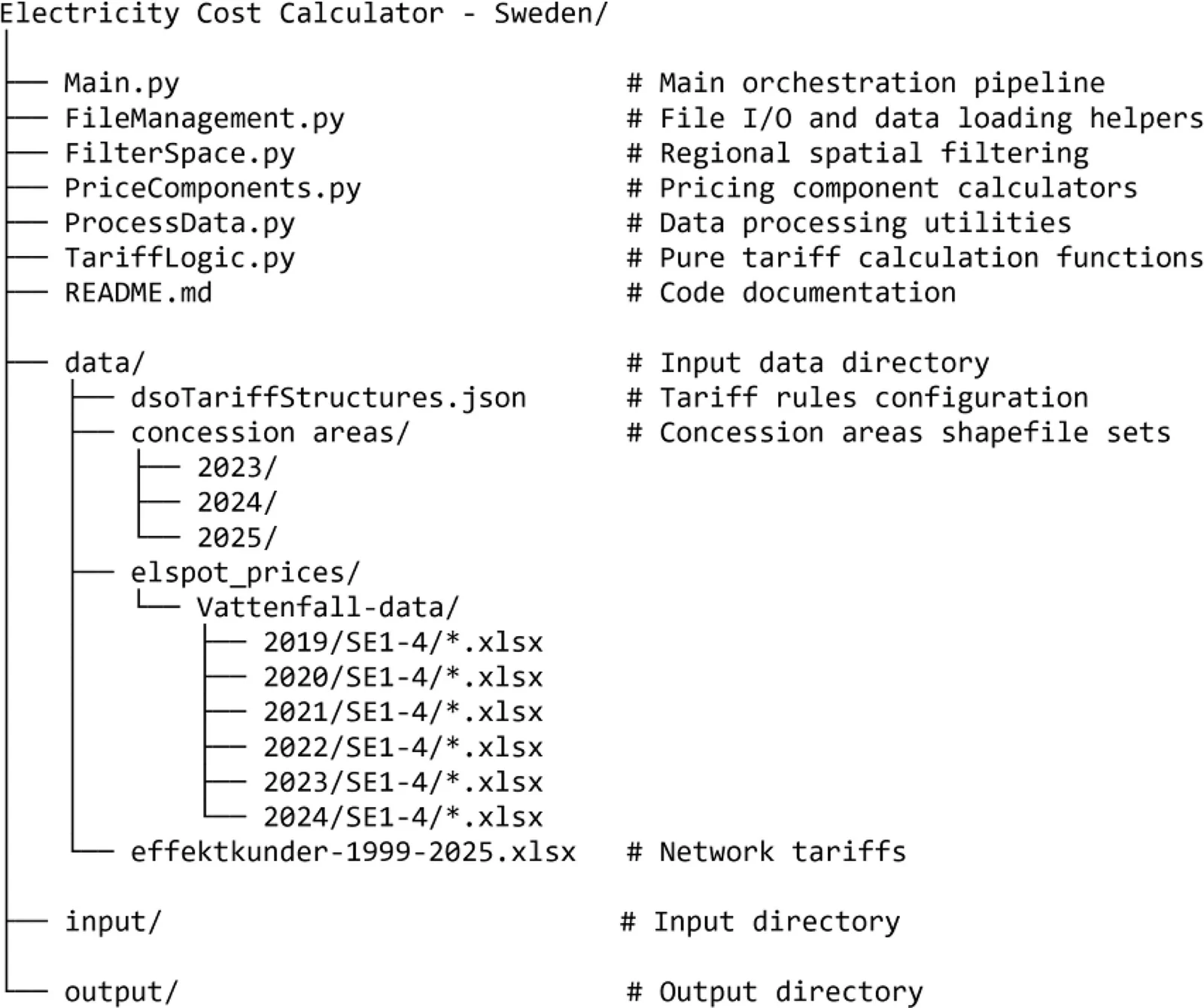
Repository structure of the Electricity Cost Calculator framework, illustrating the organization of input data, processing modules, and generated outputs.

The repository structure of the analysis toolkit is provided in Fig. 1. All raw input data (tariffs, high-load definitions, and wholesale electricity prices) are stored in the /*data* directory. The /*data* directory also contains a folder named /*concession_areas*, which includes geospatial maps of Sweden’s electricity network concession areas for 2023–2025. These maps are intended solely as reference material to assist users in determining which reporting entities (RE/REL) correspond to the geographical regions they wish to analyze. Since the concession area maps are provided only as a spatial reference to define the study area and are not used in any calculations, their temporal coverage (2023–2025) does not affect the consistency of the electricity price and tariff data, which cover the period 2019–2024. The Python modules in the root directory implement data extraction, price calculations, and multi-scenario cost analysis. All inputs to the framework (study area and load profile) must be put in the /*input* directory. All generated outputs are written to the /*output* directory.

To facilitate navigation of the repository, Table 1 summarizes the principal repository components and their roles within the workflow. Detailed documentation of file structure, module functionality, input requirements, and data formats is provided in the accompanying README file.

**Table 1:** Overview of the principal repository components, their formats and their roles within the electricity cost calculator framework. Detailed documentation of repository structure, file formats, inputs, outputs, and software modules is available in the accompanying README file.

Resource	Format	Purpose
*data/dsoTariffStructures.json*	JSON	DSO-specific tariff definitions and calculation rules
*data/concession_areas/*	GIS shapefiles	Identification of network concession areas and REL identifiers
*data/elspot_prices/*	Excel	Hourly wholesale electricity price data for SE1-SE4
*data/effektkunder-1999–2025.xlsx*	Excel	Commercial network tariff data from EI
*input/*	Excel	User-supplied study areas and load profiles
*output/*	CSV	Generated electricity cost calculations
Python modules	Python	Data processing, tariff calculations, and workflow execution
*README.md*	Markdown	Repository documentation and user guide

#### 3.1.1 Wholesale electricity pricing data

Hourly wholesale electricity prices were obtained from Vattenfall’s online price archive, which—at the time of data collection—provided publicly accessible time-series data for Sweden’s four bidding areas (SE1–SE4). The Vattenfall interface only permitted downloads on a weekly basis; consequently, the raw dataset consists of 52 Excel files per bidding area and year. These files have been preserved in the repository even though Vattenfall no longer provides free access to this historical data.

To maintain a coherent structure and reduce manual handling, the weekly Excel files are stored in a nested directory format. For each year between 2019 and 2024, four folders (SE1–SE4) contain the corresponding weekly price files. Each file includes hourly spot prices in öre/kWh (1 Swedish krona, SEK = 100 öre) along with a timestamp column. During preprocessing, the system automatically reads all weekly files for a selected year and bidding area, concatenates them into a continuous 8760-hour series (adjusted for leap years), and converts prices to SEK/MWh, which is the unit used in later cost calculations.

#### 3.1.2 Grid tariff data

The Swedish Energy Markets Inspectorate (Energimarknadsinspektionen, EI) publishes its regulatory data at the level of the *reporting entity*. In the underlying Excel dataset (*data/Effektkunder-1999–2025.xlsx*), each row corresponds to a unique reporting entity number (RE, *Redovisningsenhet*). Under Swedish regulation, a reporting entity number is a legal entity obligated to report projected investments, tariff structures, and related information to the authority. According to the *Swedish Act* (2021:311) *on Special Investment Capacity for Electricity Network Operations* (authors’ translation; Swedish: *Lag (2021:311) om särskilt investeringsutrymme för elnätsverksamhet*), a reporting entity is defined as “the network activity encompassed by a specific revenue cap.”[3] Thus, each RE number serves as a unique identifier for a DSO, irrespective of how many concession areas the operator holds. For local distribution networks, the identifier is specified as REL (*Redovisningsenhet för Lokalnät*), referring specifically to reporting entities associated with local grids.

Across the historical dataset, a DSO may have held different REL numbers over time. When a change occurs, such as if the company has been bought by a different company or EI has ordered a restructuring of accounting, EI issues a new REL number, which appears as a new row in the dataset [4]. Furthermore, prior to the early 2000s, a DSO could hold multiple REL numbers corresponding to various parts of its grid, each potentially associated with distinct tariff structures. After subsequent regulatory harmonization, each DSO is assigned a single REL number, with any variation in tariff structures instead reported through geographically defined groups. In the data processing performed in Python, the combination of the REL number and the reported group name is therefore treated as the unique identifier for a specific tariff structure.

Over the period covered by the dataset, several DSOs ceased to exist as independent entities due to mergers or acquisitions. Included in (*/data/abonnerad_effekt.csv*), is a record of DSOs that have been verified to no longer operate, together with information on the acquiring DSO where applicable.

The regulatory dataset provides information on regulatory fees (Swedish: *myndighetsavgifter*), fixed charges (Swedish: *fasta avgifter)*, subscribed capacity (Swedish: *abonnerad effekt*), high-load capacity charges (Swedish: *höglasteffekt*), and energy transmission prices during both high-load and low-load periods. These values are reported for three standardized customer types:

1. 100 kW peak demand and 350 MWh/year.
2. 1 MW peak demand and 5 GWh/year.
3. 20 MW peak demand and 140 GWh/year.

The tariff data included in this dataset cover only the capacity‑based customer categories reported by EI (Swedish: *effektkunder*). Household and small‑consumer grid tariffs are not part of this dataset and are reported separately by DSOs.

**Fig. 2:**
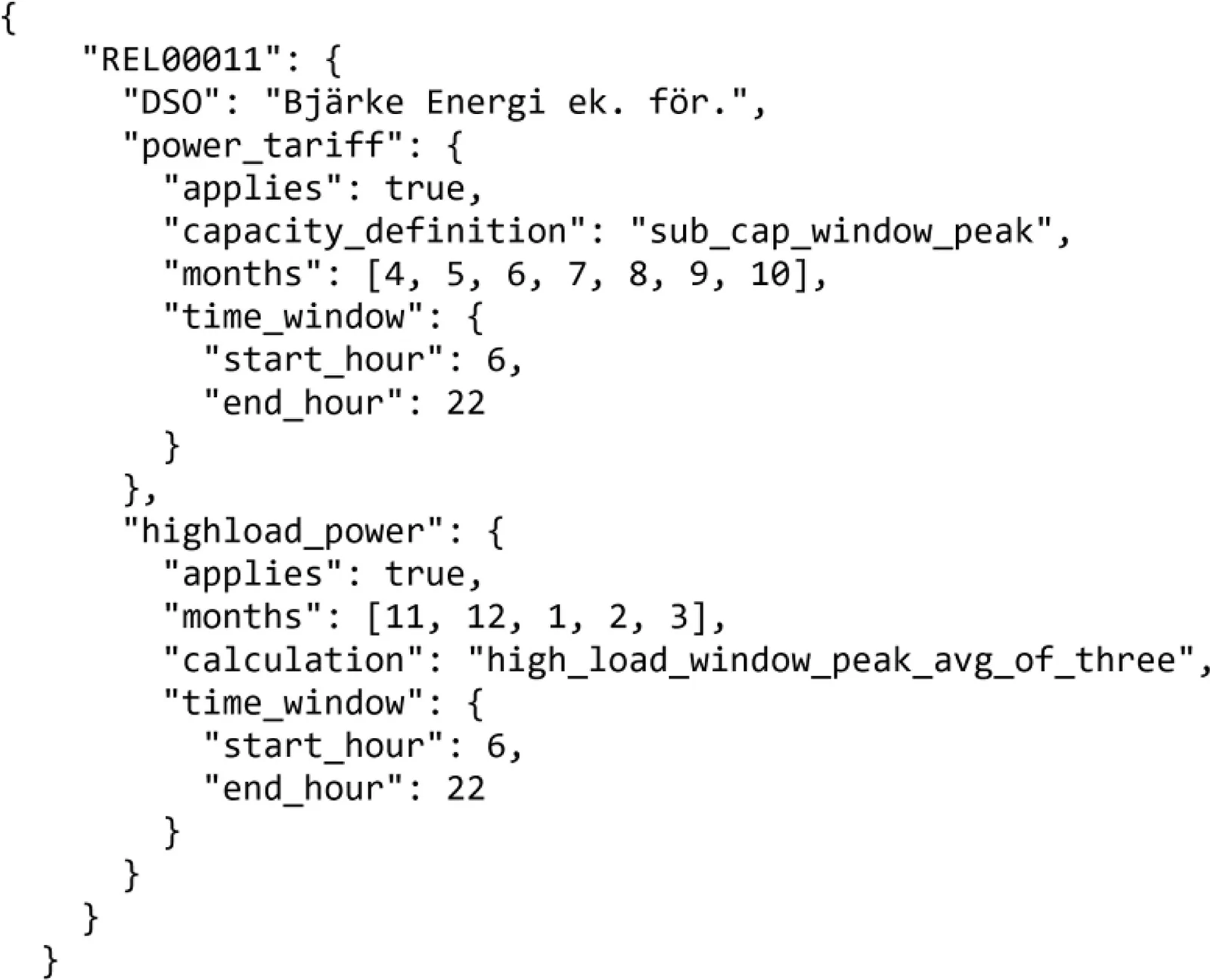
Example of the machine-readable JSON schema used to encode DSO-specific tariff rules, including subscribed-capacity definitions and high-load configurations.

The dataset does not, however, include information on how each DSO defines high-load/low-load time periods or how subscribed capacity is operationalized. DSO websites were searched manually for this information, further compiled into a JSON file (/*data/dsoTariffStructure.json*). The JSON schema follows the format illustrated in Fig. 2.

The top‑level JSON key (e.g., REL00011) serves as the unique tariff‑structure identifier and is formed as REL + GroupName (where applicable). For the RE/REL regulatory definitions, see *Grid tariff data* above. The *power_tariff* element specifies how the subscribed capacity charge is defined and applied. It details the basis for capacity determination; for example, annual peak demand or the previous month’s maximum load. The *highload_power* element describes the conditions under which high-load capacity and energy charges apply. This includes whether such a charge is used, the months during which high-load pricing is active, any specific time window defining high-load hours, the method used to identify the chargeable peak.

### 3.2 Principal data fields

Table 2 provides a summary of the principal data fields included in the dataset and processing framework. The table is intended as a quick reference for users seeking to understand the main data components, their units, and their origin within the workflow.

**Table 2:** Principal data fields used in the dataset and electricity cost calculation framework, including variable descriptions, units, and data sources.

Variable	Description	Unit	Source
Wholesale price	Hourly day-ahead electricity price for the selected bidding area	Öre/kWh (raw), SEK/MWh (processed)	Vattenfall
REL	Reporting entity identifier for local grid tariff structure	-	EI
GroupName	Geographic tariff subgroup where applicable	-	EI
Fixed fee	Fixed network tariff component	SEK/year	EI
Energy charge	Consumption based network tariff component	SEK/MWh	EI
Subscribed capacity	Capacity level used for power-based billing	kW or MW	EI + DSO documentation
High load charge	Additional charge during high-load periods where applicable	SEK/kW	EI + DSO documentation
High load months and/or hours	Months and hours included in high load calculations where applicable	Month index	DSO documentation
Load (kWh)	Hourly electricity demand profile supplied by user	kWh	User input

## 4 Experimental Design, Materials and Methods

### 4.1 Software and environment

The framework is implemented in Python (3.8+), using pandas, numpy, openpyxl, and standard library modules for time and file handling. The repository includes modular scripts for ingestion, transformation, and cost calculation. The computational workflow has been modularized into five dedicated Python modules—FileManagement, FilterSpace, ProcessData, TariffLogic, and PriceComponents—to separate data ingestion, spatial filtering, load-profile handling, tariff logic, and price-component assembly.

### 4.2 Data acquisition

#### 4.2.1 Wholesale prices

For 2019–2024, hourly price data were sourced from Vattenfall [5] as Excel files for each bidding area (SE1–SE4) and stored under */data/elspot_prices/Vattenfall-data/{YEAR}/{AREA}/*. During processing, files for the selected year/area are concatenated into a continuous annual series. During preprocessing, timestamps are standardized to a consistent 8760-hour annual structure through automatic removal of February 29 in leap-years and appropriate handling of daylight-saving transitions. All electricity prices were converted from öre/kWh to SEK/MWh.

#### 4.2.2 Grid tariffs

Tariff data were imported from EI’s multi‑indexed Excel workbook [6] and stored under */data/effektkunder-1999–2025.xlsx* at the reporting‑entity level (RE/REL). All tariff columns were retained without modification; rows sharing the same REL + GroupName combination were merged into a single record. EI’s dataset sometimes contains multiple rows for the same reporting entity when tariff information is spread across year‑specific columns; however, values never overlap across these duplicate rows. The processing pipeline therefore consolidates them into one unique key per tariff structure to ensure unique identifiers.

Tariff attributes not present in the EI regulatory workbook—such as high-load months, high-load hourly windows, and capacity-definition rules—are stored in a machine-readable JSON file (*dsoTariffStructures.json*) and linked to the tariff table using the REL or REL+GroupName identifiers. The data was gathered during the period of February 18 – March 7, 2026.

### 4.3 Python modules

#### 4.3.1 Main.py — main analysis

The core of the computational framework. It sets up the workflow: loading input data, constructing hourly load profiles for each scenario, integrating network tariff parameters, retrieving hourly wholesale electricity prices, and computing all cost components. The script contains a main() function used for running the analysis. It outputs three datasets summarizing (i) the full hourly cost breakdown, (ii) cost redistribution for 24-hour visualization, and (iii) the fully expanded load profiles.

#### 4.3.2 FileManagement.py — input/output and data retrieval

All reading of external data sources is in this module. It contains dedicated functions to load wholesale electricity prices (weekly Vattenfall files concatenated into annual series), network tariff data from The Swedish Energy Market Inspectorate, regional study-area configurations, and 24-hour load profiles.

#### 4.3.3 FilterSpace.py — spatial filtering of DSOs and regions

Manages the spatial dimension of the analysis. It filters the full tariff dataset to the specific concession areas included in the study, ensuring that only relevant DSOs and reporting entities are used in the cost calculations.

#### 4.3.4 ProcessData.py — data transformation utilities

Contains supporting functions for internal data structuring, such as cleaning intermediate data frames, aligning tariff tables with JSON-based rule definitions, and preparing data formats for the main cost analysis.

#### 4.3.5 dsoTariffStructures.json — JSON tariff rules

Configurable JSON file that provides per-DSO rule definitions for subscribed capacity and high-load periods. These rules determine how peak power is calculated, which months count as high-load, and what hourly windows apply. The calculator reads this file at runtime, enabling the analysis to reflect DSO-specific tariff structures that are not explicitly present in the Excel report.

### 4.4 Required user inputs

#### 4.4.1 Load scenarios

**Fig. 3:**
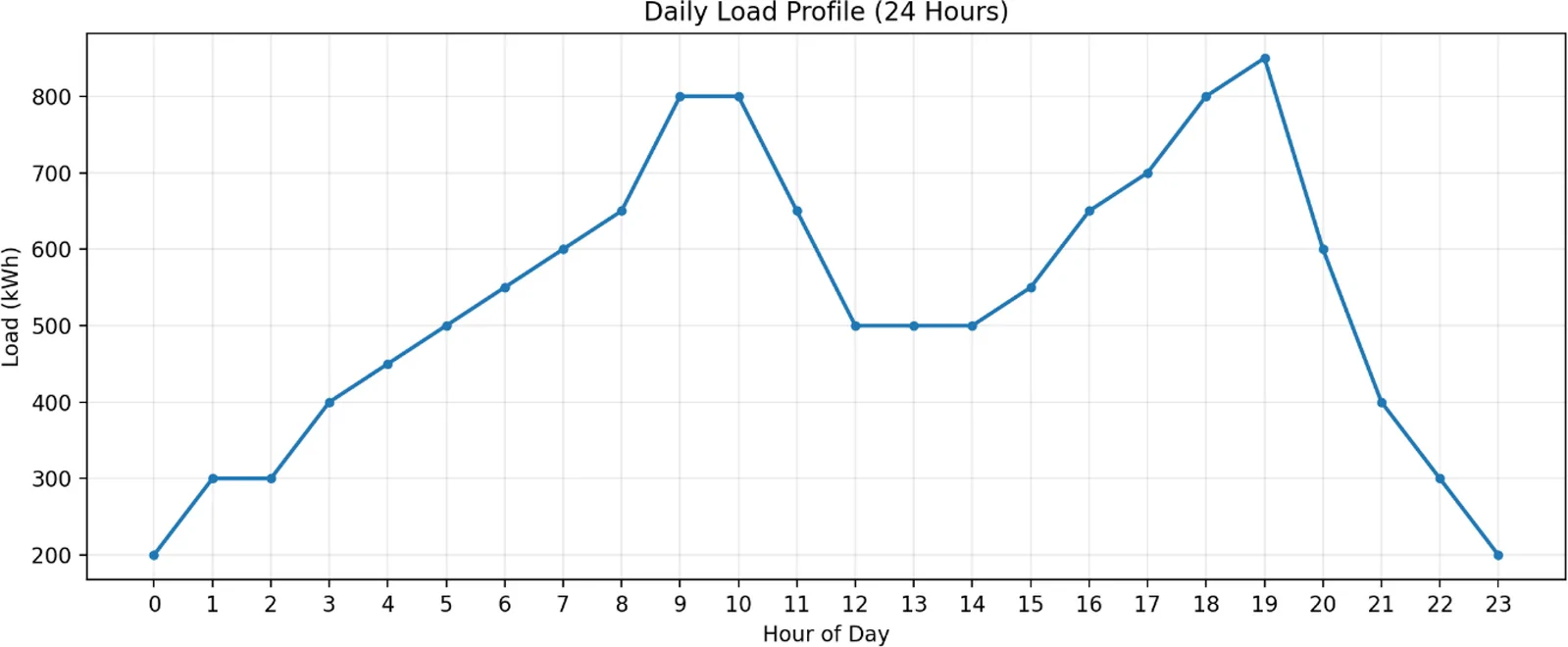
Example 24-hour load profile used as input to the electricity cost calculator. Such profiles are automatically expanded to annual resolution within the processing framework.

Since electricity prices are dependent on the timing of energy and power consumption, a load profile is needed to get costs - users must upload their own load profiles in Excel format (an example is visualized in Fig. 3).

The system either expands user-supplied 24-hour profiles to full-year resolution (8760 hours) or accepts full 8760-hour profiles and applies seasonal and weekday/weekend differentiation. The framework expects an excel file with the load data under a column called *Load (kWh)*. These profiles are processed through the same pipeline to calculate electricity costs under different tariff structures.

#### 4.4.2 Study area

Users must specify which distribution network areas to include in the analysis. Because total electricity prices depend on the local distribution system operator (DSO), the framework requires a list of REL identifiers (and, if applicable, REL+GroupName keys when a DSO reports multiple tariff structures). Users also select the year they wish to model. The study area must be provided as an Excel file following the example format shown in Table 3. For regulatory definitions of RE/REL identifiers, see the *Grid tariff data* section.

**Table 3:** Example study area definition showing DSO identifiers, reporting entities (REL), and corresponding electricity bidding areas.

DSO (long)	DSO (short)	RE + subgroup (2024)	Bidding Area
Brittedals Elnät ek. för.	DSO 11	REL01012	SE3
Bromölla Energi och Vatten AB	DSO 12	REL00021	SE3
C4 Elnät AB	DSO 13	REL00023	SE3
Dala Energi Elnät AB	DSO 14	REL03009	SE3
Eksjö Elnät AB	DSO 15	REL00030	SE3
Ellevio AB	DSO 16	REL03035Mellansverige	SE3
Ellevio AB	DSO 17	REL03035Edsbyn	SE3
Ellevio AB	DSO 18	REL03035Västkusten	SE3
Emmaboda Elnät AB	DSO 19	REL00031	SE3

**Fig. 4:**
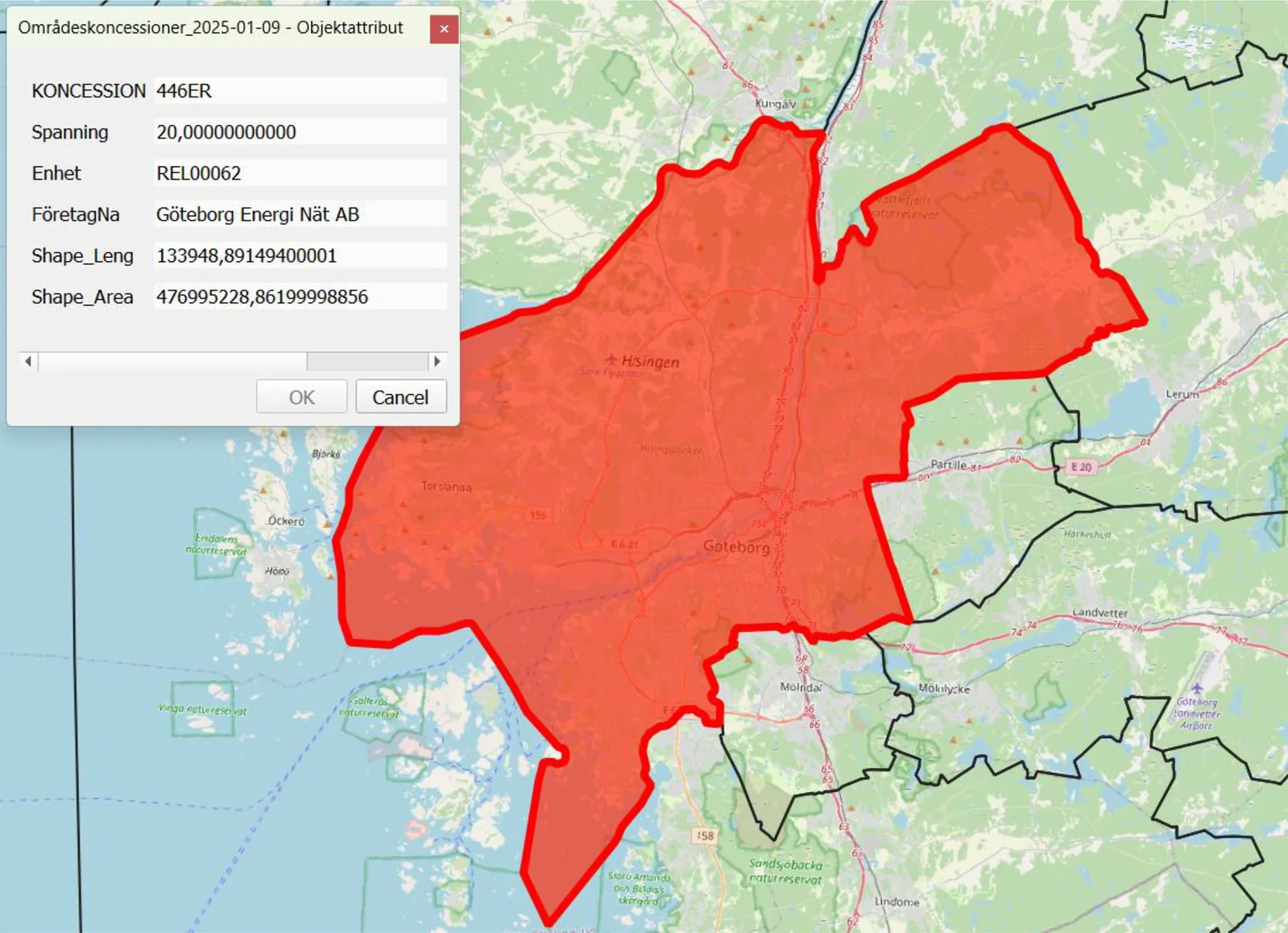
Example concession area map included in the repository illustrating the relationship between geographic concession areas, DSOs and REL identifiers. The example shows the areas surrounding Gothenburg, where the highlighted concession area is operated by Göteborg Energi Nät and associated with reporting entity REL00062.

To support the correct selection of reporting entities for the study area, the repository includes a set of GIS maps under */data/concession_areas*. These maps depict all Swedish electricity network concession areas for 2023–2025 and are provided as a visual aid for identifying which REL numbers correspond to the geographical area of interest. While these files are not used in any computational step of the workflow, they ensure that users can more easily verify the spatial boundaries of DSOs before specifying their study area. Fig. 4 provides an example of the information contained within these maps. Given a geographic area of interest, users can identify the corresponding concession area, DSO, and REL identifier required as input to the framework.

### 4.5 Example workflow execution

A minimal working example of the workflow is as follows:

1. Provide a load profile (24-hour or 8760-hour format) in the */input* directory
2. Define the study area by specifying REL identifiers and bidding areas
3. Set parameters in *Main.py* (customer type, year, input files)
4. Run the script to generate hourly and aggregated cost outputs

A complete step-by-step guide, example input files, configuration parameters and documentation of all modules are provided in the accompanying *README* file available through the GitHub repository.

**Fig. 5:**
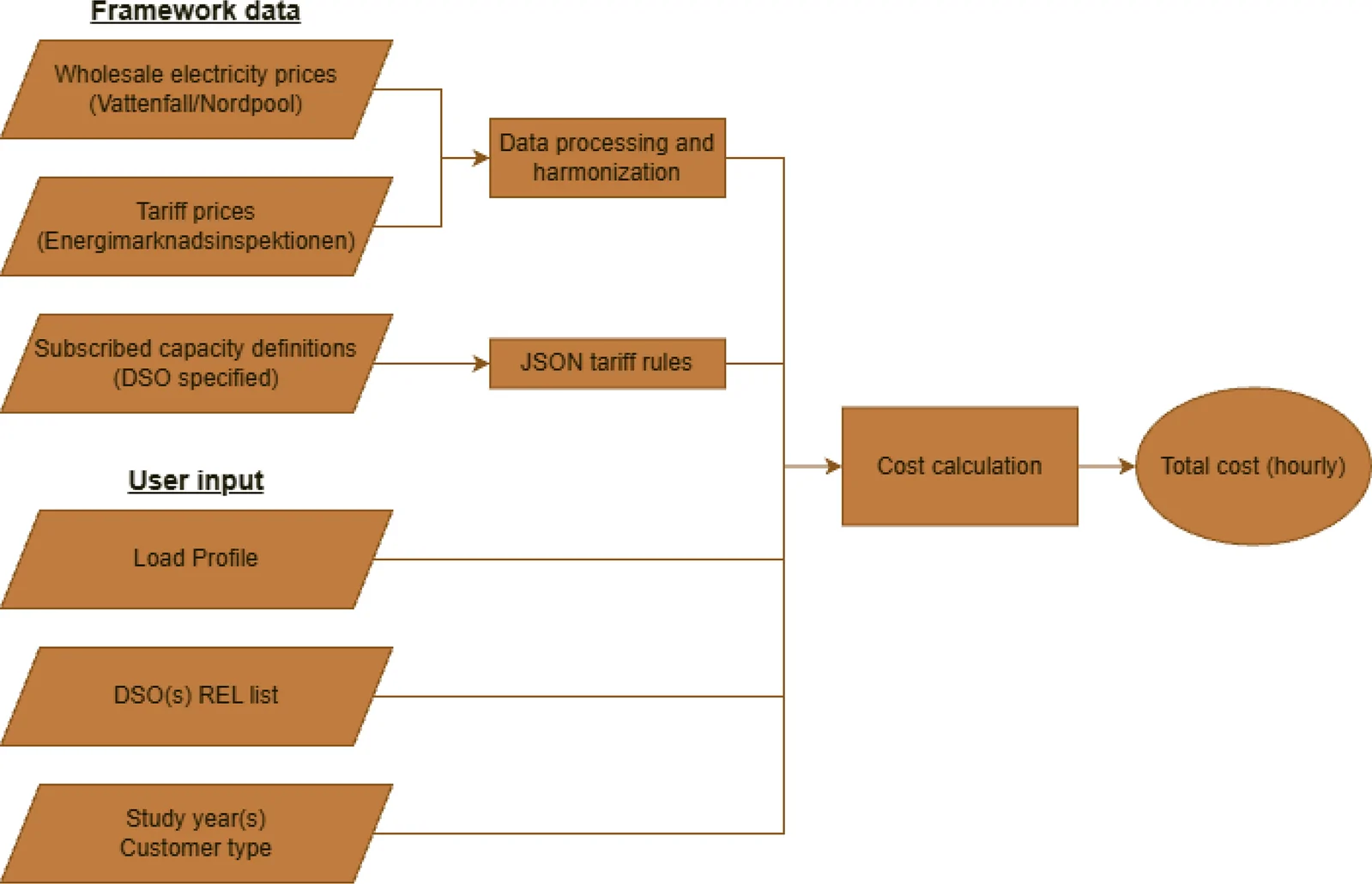
Overview of the processing workflow used to combine wholesale electricity prices, regulatory tariff data, DSO-specific tariff definitions, and user-defined load profiles into hourly electricity cost estimates.

**Fig. 6:**
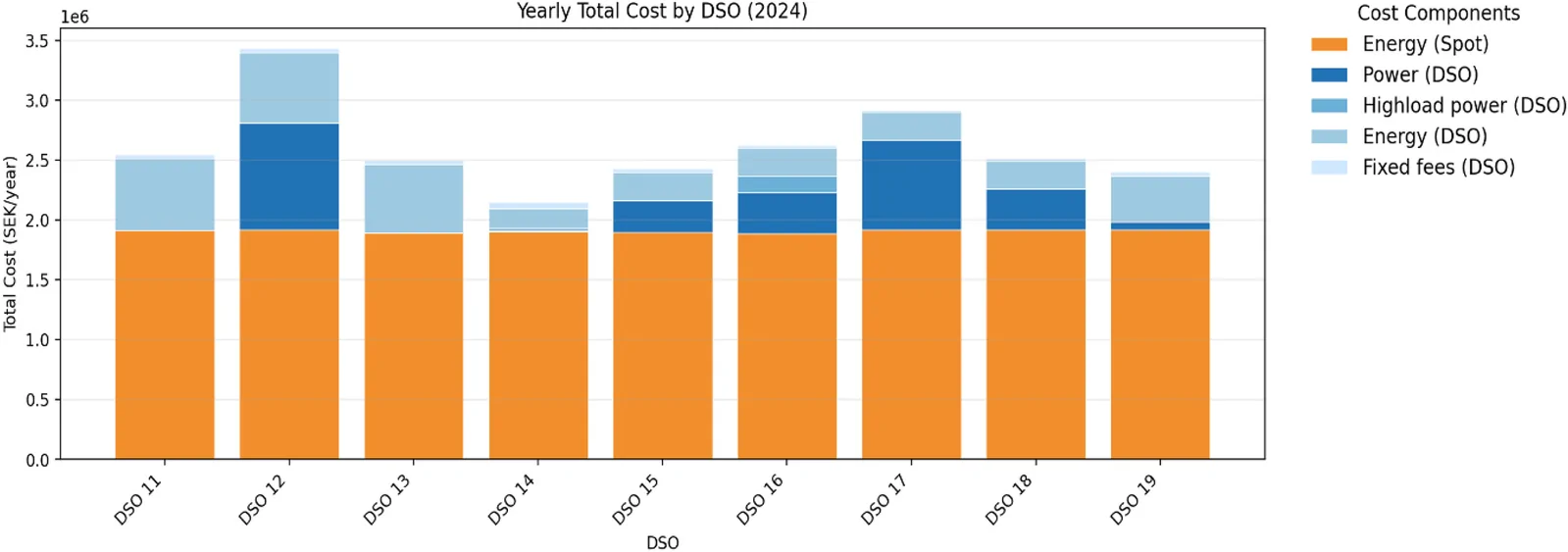
Example analysis output from the electricity cost calculator. Hourly electricity costs have been aggregated to annual resolution, and the resulting yearly costs are shown for all DSOs included in the study area. Cost components are displayed as a stacked bar chart, illustrating the contribution of different tariff and electricity price elements to the total annual cost.

The framework preserves results at hourly resolution, allowing users to aggregate, analyze, and visualize outputs according to the requirements of their specific research questions. Using the example load profile in Fig. 3 and the example study area defined in Table 3 users can reproduce the results presented in Fig. 6 by executing the workflow illustrated in Fig. 5 with the supplied example inputs.

Fig. 6 illustrates one possible aggregation of the hourly output data. Users can alternatively analyze results at hourly, daily, monthly, seasonal, or annual resolution depending on their research objectives.

### 4.6 Data provenance and source retrieval

Table 4 summarizes the primary external data sources used to construct the dataset, together with their temporal coverage and retrieval periods. The table is intended to support transparency and reproducibility by documenting the provenance of the data incorporated into the repository.

**Table 4:** Primary external data sources used to construct the dataset, including their content, temporal coverage, and retrieval periods.

Source	Content	Coverage	Retrieval date	Update frequency
Vattenfall price archive	Hourly wholesale electricity prices	2019–2024	2026–02–25	Historical (no longer updated)
Swedish Energy Markets Inspectorate (EI)	Commercial network tariffs (capacity-based customers)	1999–2025	2026–02–25	Annual
DSO websites and tariff documentation	High-load definitions, capacity rules, tariff logic	Current definitions	2026–02–18 to 2026–03–07	Variable, typically annually

For reproducibility, the processed versions of these data sources are archived within the repository and linked to the software workflow described in this article. Users extending the dataset should document the retrieval date of any newly acquired source material and verify consistency with previous datasets.

### 4.7 Future updates and version management

The dataset and associated Python framework were designed to support future updates as new electricity tariff and wholesale price data become available. Future tariff updates can be incorporated through the following workflow:

1. Download the latest tariff workbook and concession area maps from the Swedish Energy Markets Inspectorate (EI).
2. Identify any changes to reporting entities (RE/REL identifiers), including mergers, acquisitions, renaming, or modifications to group-based tariff reporting. Comparison with concession areas from previous years may be required to determine which concession areas are affected. Geographic Information System (GIS) software can be used to support this process.
3. Review DSO documentation and websites for changes to subscribed-capacity definitions, high-load periods, or other tariff rules not reported by EI. This step requires manual review of the relevant DSO websites and associated tariff documentation. Links to DSO websites are provided in the file *abonnerad_effekt.csv* located in the */data* directory. This file was created as a supporting artefact during compilation of *dsoTariffStructures.json* and is included solely as a reference source for website locations; it does not need to be updated when revising the JSON tariff definitions.
4. Update the corresponding entries in *dsoTariffStructures.json* to reflect any identified changes in tariff structures.
5. Acquire updated wholesale electricity price data and store them using the same directory structure and unit conventions described in this article.
6. Validate the updated dataset by executing the supplied workflow and confirming that tariff structures are correctly linked to the corresponding DSO identifiers and produce expected outputs.

The authors intend to update the tariff and wholesale electricity price data periodically as resources permit. Information regarding the most recent verified dataset release and data coverage will be documented in the repository README. To ensure consistency and quality control, updates to the official dataset are maintained by the repository authors. Users wishing to extend or adapt the dataset are encouraged to do so through local copies of the repository rather than modifying the archived release. Stable dataset releases are archived through Zenodo, while the GitHub repository provides access to the most recent documentation and software implementation. However, no commitment can be made regarding the frequency or long-term commitment of future official updates.

## Limitations

The framework is designed for Swedish electricity markets and reflects country-specific tariff structures and regulatory reporting formats. While the framework is designed to be extensible, it is not directly transferable to other countries by simply substituting new tariff and price datasets. Network tariff structures, regulatory reporting systems, capacity definitions, and peak-demand charging mechanisms vary considerably between national electricity markets. Applying the framework outside Sweden would therefore require both new input data and modifications to the tariff logic to reflect country-specific pricing rules. The wholesale price inputs were obtained from a historical download interface that exported weekly Excel files; the repository includes only 2019–2024. Source availability has changed over time, which may affect future updates; users may need to substitute alternative sources while preserving the folder and unit conventions described in this article. Tariff data reflect EI’s reporting‑entity (RE/REL) structure; nomenclature and groupings can change over time (e.g., mergers, renaming, and group‑based reporting), requiring careful key maintenance when extending the dataset. While the JSON-based tariff rule system captures DSO-specific logic for 2025, users extending the dataset to earlier or later years must update these rule files manually to match any changes in DSO tariff definitions. High‑load months/hours and capacity definitions are not historical, but instead only represent the definitions as stated for the year 2025. These can change over time, requiring consideration and maintenance when extending the dataset. All currency values are preserved as nominal in the year of record. The dataset reflects current tariff structures and hourly wholesale prices but does not include anticipated changes such as 15-minute spot that Nordpool has used since September 30, 2025, or experimental time-based capacity tariffs that some DSOs may introduce in the future. These limitations should be considered when applying the dataset to projections or future policy analysis.

## Ethics Statement

The authors confirm that this work does not involve human subjects, animal experiments, or data collected from social media platforms. The authors have read and follow the ethical requirements for publication in *Data in Brief*.

## CRediT Author Statement

**Gabriella Grenander:** Conceptualization, methodology, software, data curation, formal analysis, writing – original draft, writing – review & editing, visualization. **David Daniels:** Project administration, validation, writing – review & editing, resources, discussion of scope and content.

## Declaration of Generative AI and AI-assisted Technologies in the Manuscript Preparation Process

During the preparation of this work the author(s) used Copilot to assist in editing the text. After using this tool/service, the author(s) reviewed and edited the content as needed and take(s) full responsibility for the content of the published article.

## Data Availability

GithubElectricity_Cost_Calculator-Sweden (Original data).
